# Supplementary material for: Divergent trajectories to structural diversity impact patient survival in high grade serous ovarian cancer
Source: Nat Commun. 2025 Jul 1;16:5586. doi: 10.1038/s41467-025-60655-y (PMC12215056; doi:10.1038/s41467-025-60655-y)
Supplement: Supplementary file 1 — Supplementary Information [file 41467_2025_60655_MOESM1_ESM.pdf]

Divergent trajectories to structural diversity impact patient survival in high grade serous ovarian cancer

Ailith Ewing*, Alison Meynert, Ryan Silk, Stuart Aitken, Devin P. Bendixsen, Michael Churchman, Stuart L. Brown, Alhafidz Hamdan, Joanne Mattocks, Graeme R. Grimes, Tracy Ballinger, Robert L. Hollis, C. Simon Herrington, John P. Thomson, Kitty Sherwood, Thomas Parry, Edward Esiri-Bloom, Clare Bartos, Ian Croy, Michelle Ferguson, Mairi Lennie, Trevor McGoldrick, Neil McPhail, Nadeem Siddiqui, Rosalind Glasspool, Melanie Mackean, Fiona Nussey, Brian McDade, Darren Ennis, The Scottish Genomes Partnership, Lynn McMahon, Athena Matakidou, Brian Dougherty, Ruth March, J. Carl Barrett, Iain A. McNeish, Andrew V. Biankin, Patricia Roxburgh#, Charlie Gourley#, Colin A. Semple#.

#Equal contribution
*Corresponding author: ailith.ewing@ed.ac.uk

Supplementary Information

Supplementary Figures 1-13
Supplementary References
Scottish Genomes Partnership Consortium members and affiliations

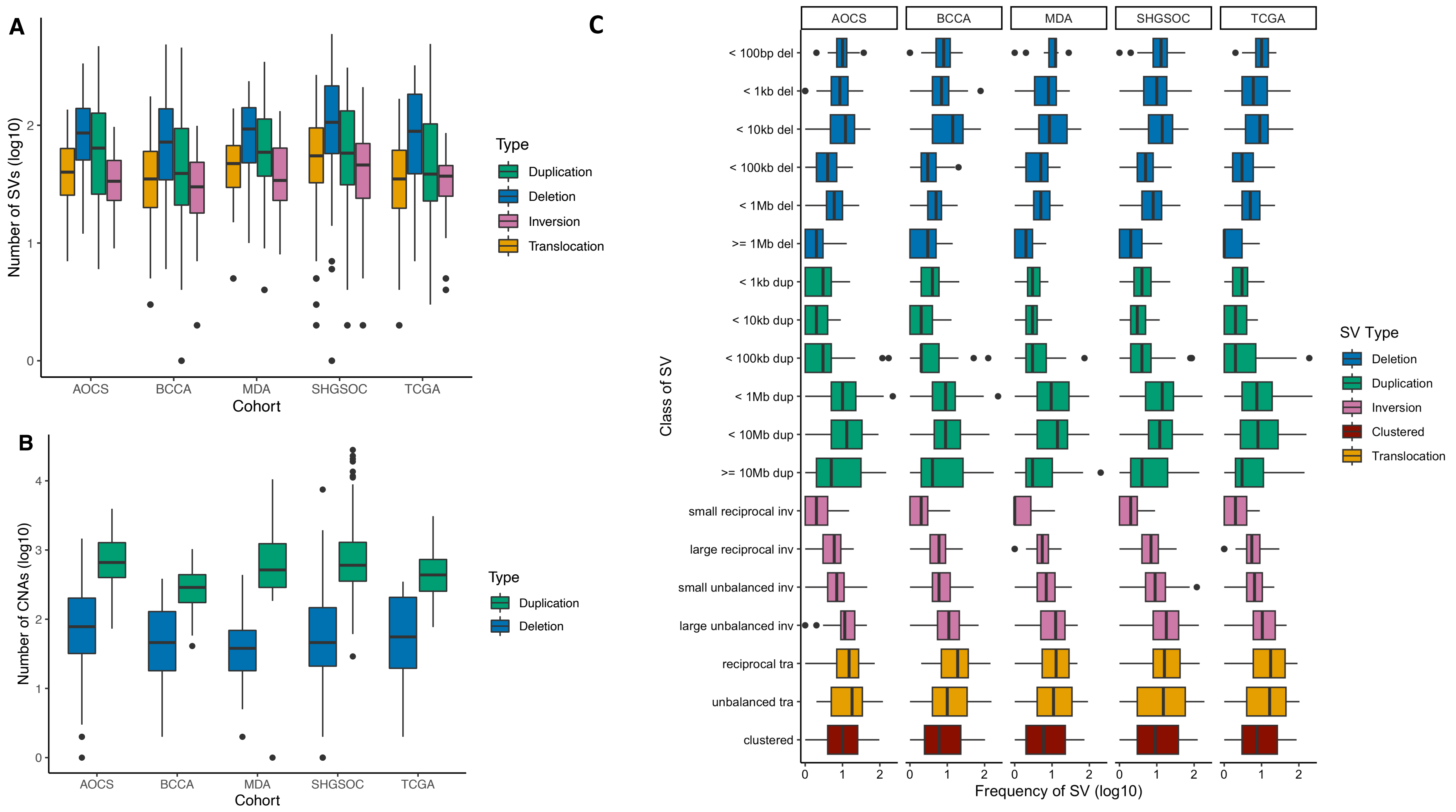

Supp Figure 1: SV and CNA burdens and genomic spans are broadly consistent across AOCS (N=80) BCCA (N=59), MDA (N=26), SHGSOC (N=115) and TCGA (N=44) cohorts. (A) The numbers (log10) of consensus deletion, duplication, inversions and translocations by cohort. (B) The numbers (log10) of consensus deletion and duplication CNAs across cohorts. (C) The numbers (log10) of SVs by length and type across cohorts. All box plots show the data median (centre), 25% and 75% percentiles (box boundaries), dispersal (whiskers indicating 1.5 times the interquartile range) and outliers (points). (Somatic variant calls encompassing SNV, SV and CNA for all samples are provided in Source Data.)

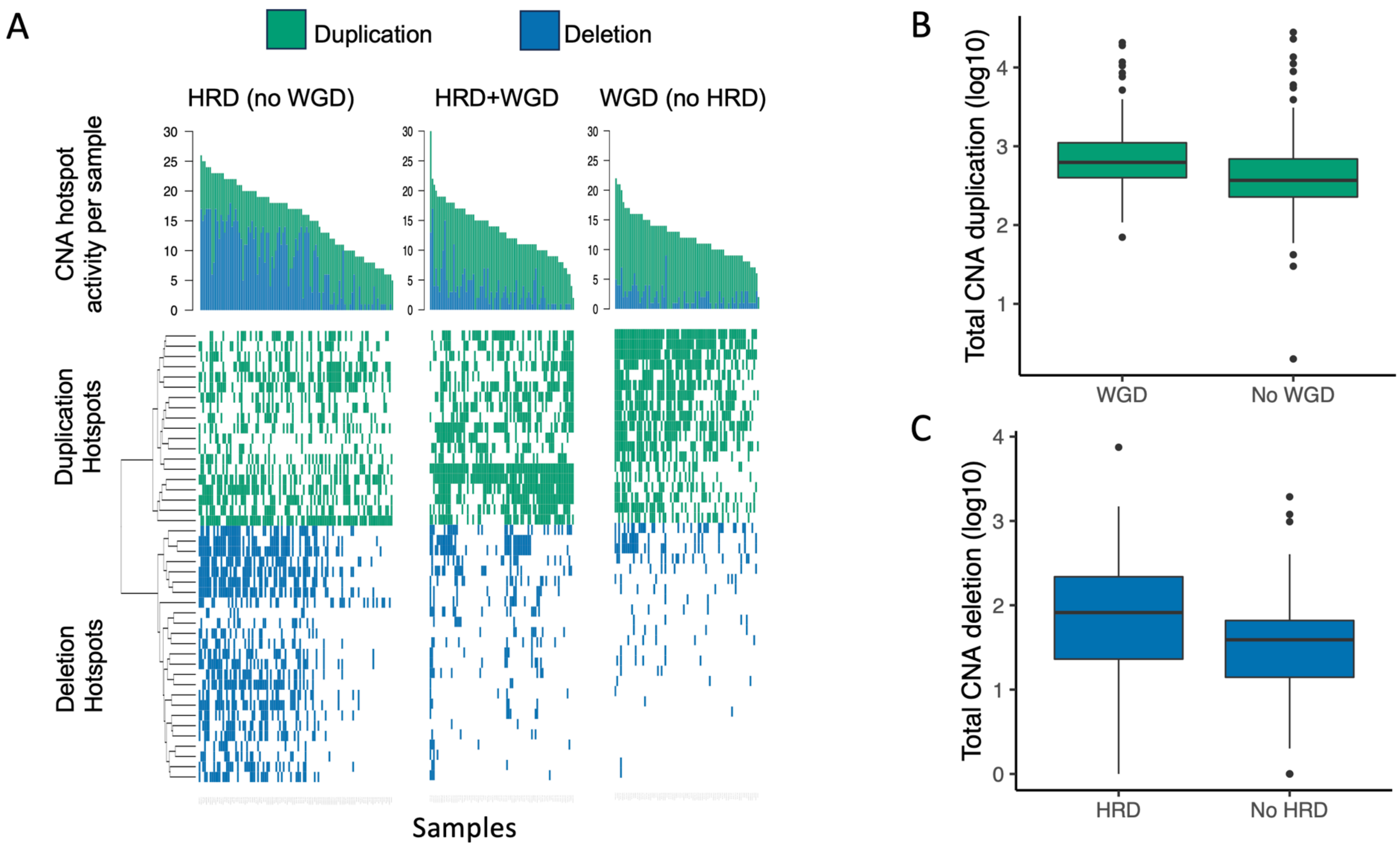

Supp Figure 2: CNA burdens in hotspots and genome-wide by WGD and HRD. (A) CNA hotspot activity varies across samples with greater activity at deletion hotspots in HRD samples (N=181) and greater duplication activity in WGD samples (N=158). (B) WGD samples have significantly higher duplication rates genome-wide per sample than samples lacking WGD (Wilcoxon $p<1.8x10^{-8}$); (C) HRD samples have significantly higher deletion rates than samples lacking HRD (Wilcoxon $p<2.8x10^{-5}$). All box plots show the data median (centre), 25% and 75% percentiles (box boundaries), dispersal (whiskers indicating 1.5 times the interquartile range) and outliers (points). HRD, WGD and CNA hotspot data are in Tables S1 and S8 (Supplementary Dataset 1) respectively.

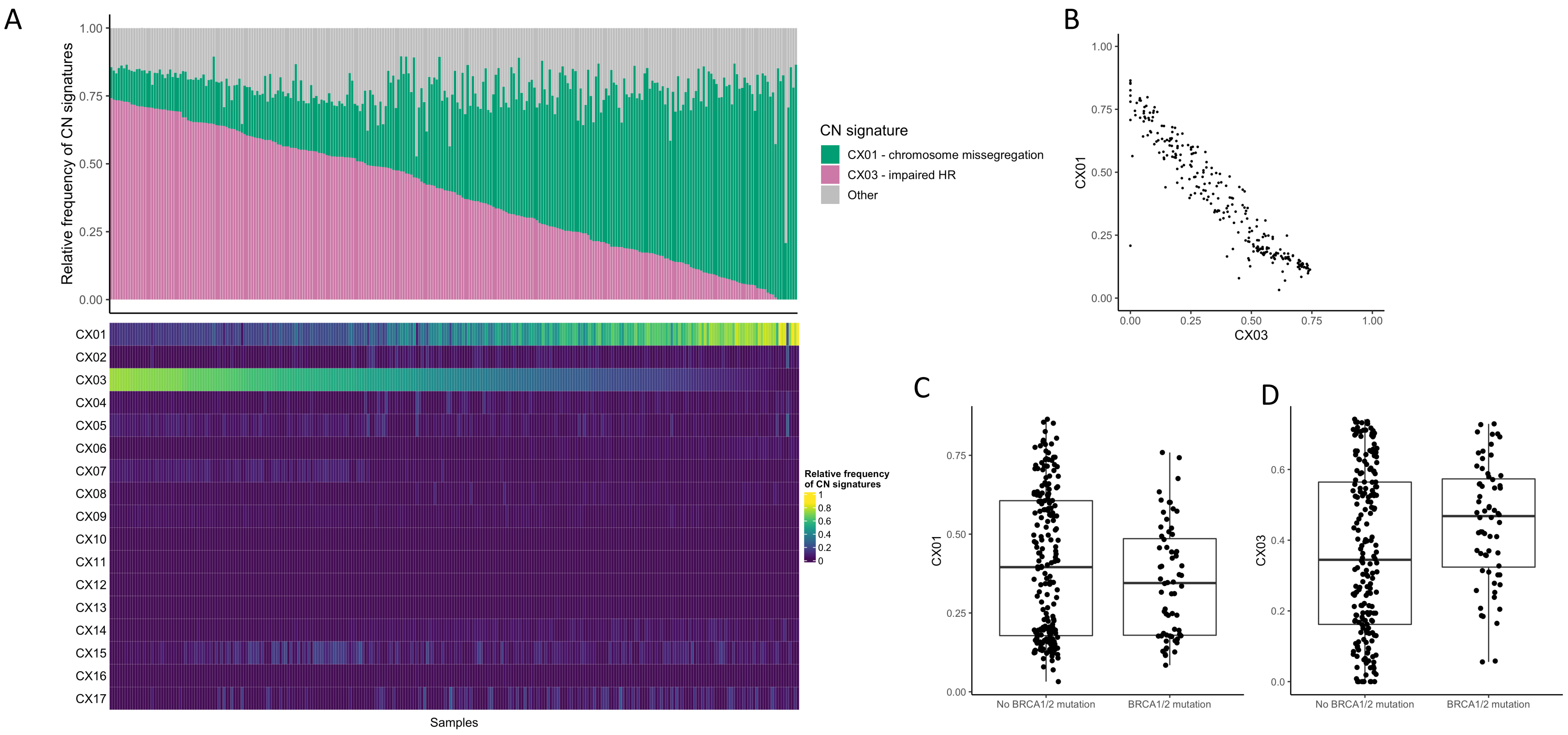

Supp Figure 3: CNA signature exposures reflect scars of impaired HR and chromosome missegregation. (A) Relative frequencies of exposures of known CN signatures[1] across samples are dominated by signatures CX01 and CX03. (B) Exposures for CX01 and CX03 are strongly inversely correlated. (C) Exposures of CX01 in BRCA1/2 mutated and non-BRCA1/2 samples. (D) Exposures of CX03 in BRCA1/2 mutated and non-BRCA1/2 samples. CX03 exposure significantly higher in BRCA1/2 mutated samples (p=0.01). Only samples with > 40% cellularity (N = 268) were included as recommended by Drews et al[1]. All box plots show the data median (centre), 25% and 75% percentiles (box boundaries), dispersal (whiskers indicating 1.5 times the interquartile range) and outliers (points). Signature exposure data are in Table S4 (Supplementary Dataset 1).

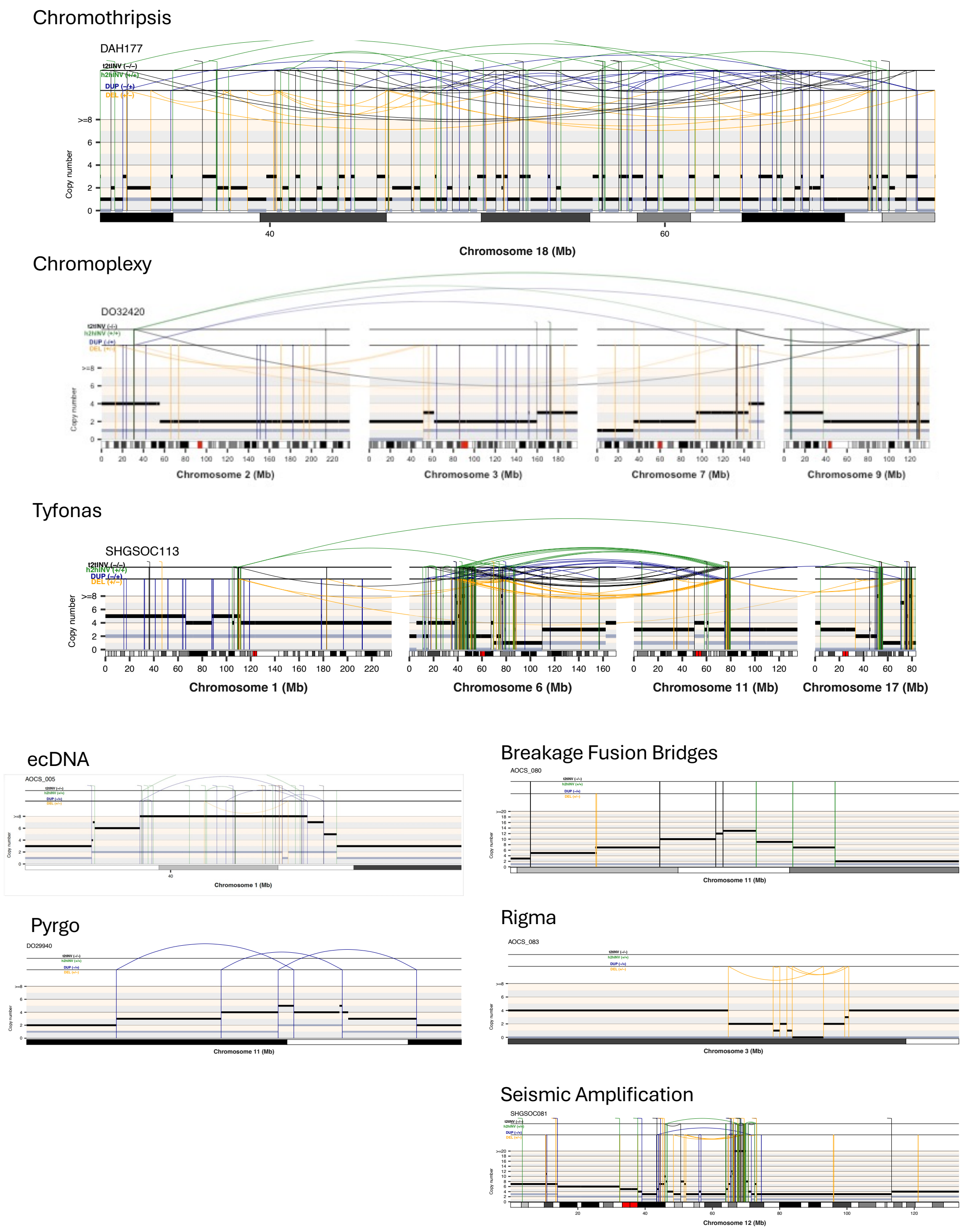

Supp Figure 4: Visual examples of the eight types of complex SVs characterized. Chromothripsis, chromoplexy, tyfonas, ecDNA, breakage-fusion bridges, pyrgo, rigma and seismic amplification. Figures generated using consensus SV and CNV calls using R package RConPlot[2]. All complex SV data are in Table S1 (Supplementary Dataset 1).

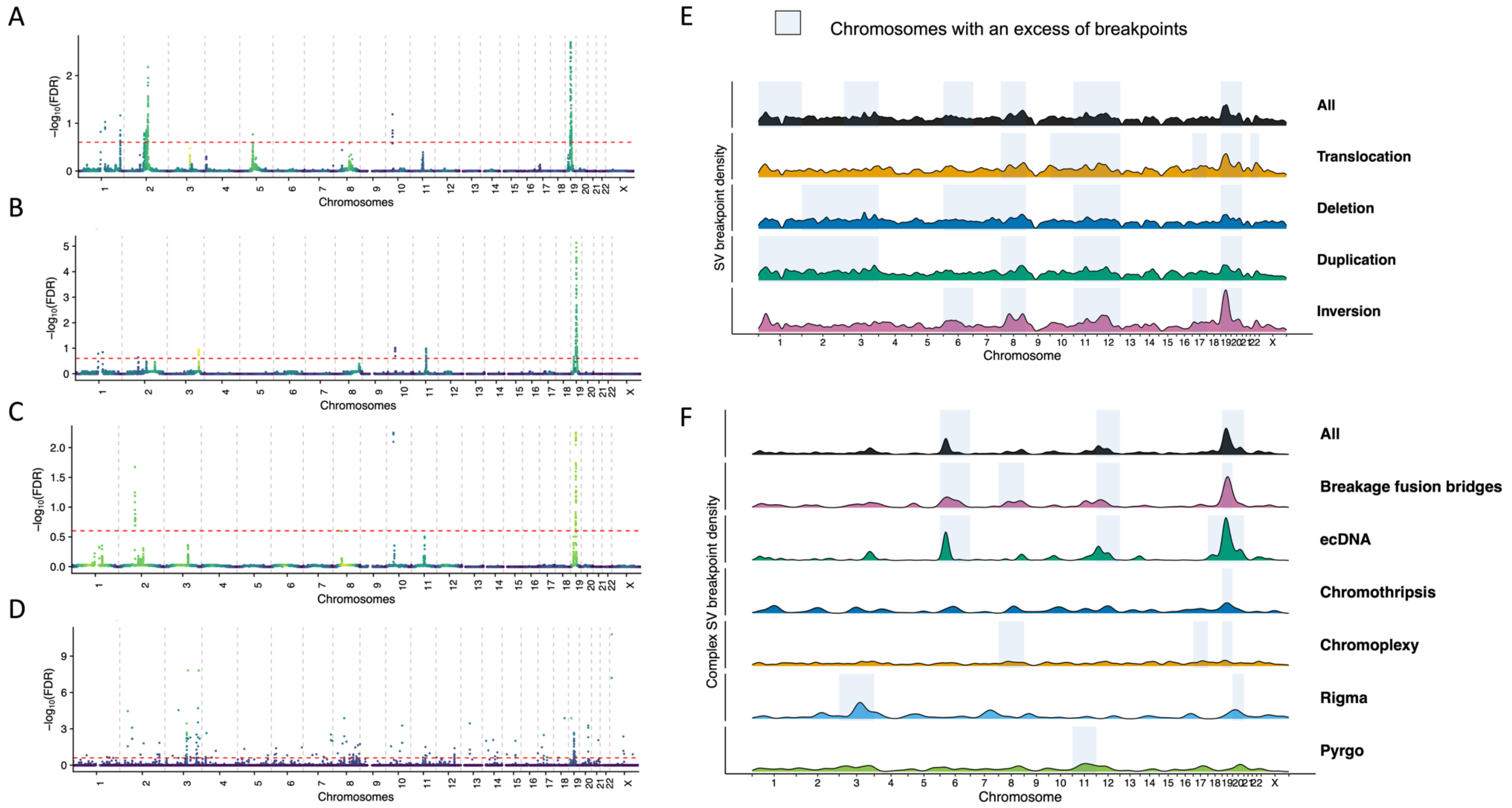

Supp Figure 5: Enrichment of breakpoints associated with SVs and complex SVs across the combined cohort (N=324). (A-D) Genomic regions recurrently impacted by individual SV types across the cohort based upon FishHook analysis of consensus SV data in 50Kb windows across the genome (Methods), for (A) recurrent deletions, (B) duplications, (C) inversions and (D) breakpoints. The red lines indicate FDR < 0.25 threshold which is far lower than the height of the peaks. (E) Chromosomal enrichment of SV breakpoint density throughout the genome for all classes of SV. (F) Breakpoint density of SVs associated with complex SVs throughout the genome by complex SV type. Light blue-coloured chromosomes have a significant excess of breakpoints adjusted for chromosome size (binomial test of proportions, $p < 0.05$). Y-axis scales are consistent across SV types within each panel. Hotspot and chromosome enrichment data are in Tables S7 and S5 (Supplementary Dataset 1) respectively.

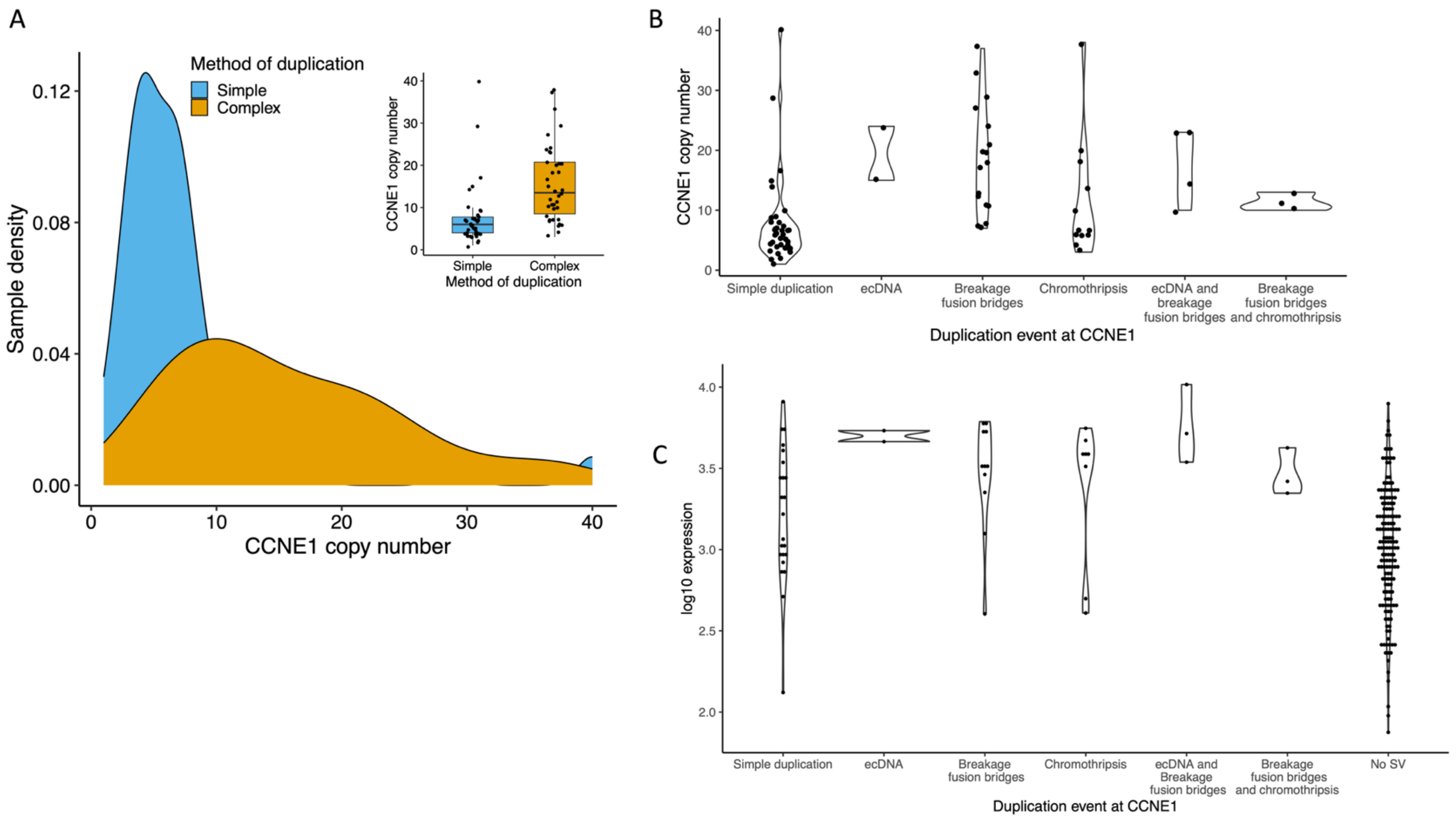

Supp Figure 6: Amplification of *CCNE1* by simple SVs and cSV types. (A) The copy number of *CCNE1* amplified by complex SVs or simple duplication differs significantly (Wilcoxon p = 2.19×10^{-6}). *CCNE1* is amplified in 22% (N=71) of samples by simple and complex SV types (Table S6, Supplementary Dataset 1). Amplification by simple SV generally results in lower copy numbers. (B) Copy number of *CCNE1* amplified by simple SV events and observed combinations of cSV. All combinations of complex SV mediated amplification are associated with a higher degree of amplification. (C) Expression of *CCNE1* amplified by simple SV events and observed combinations of cSV. All combinations of complex SV mediated amplification are associated with higher differential *CCNE1* expression. All box plots show the data median (centre), 25% and 75% percentiles (box boundaries), dispersal (whiskers indicating 1.5 times the interquartile range) and outliers (points). CCNE1 differential expression data are in Table S6 (Supplementary Dataset 1).

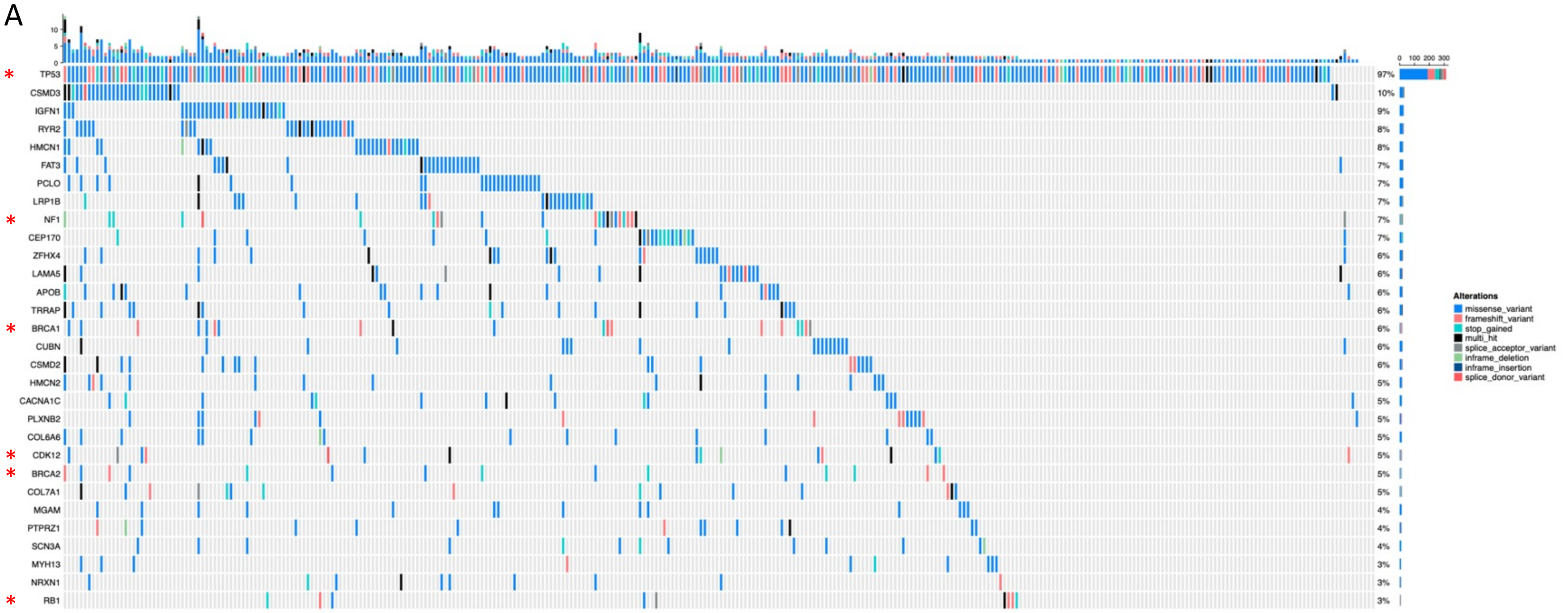

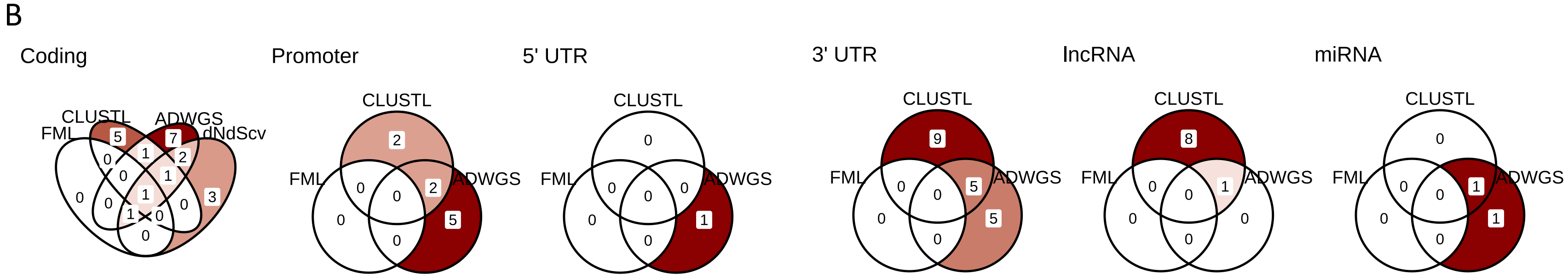

Supp Figure 7: Recurrently SNV altered genes and candidate driver variants. (A) SNV oncoplot (corrected for gene length) indicating protein coding genes subject to recurrent SNVs across the cohort and predicted to be driver genes (red stars) by dNdSCV (Methods). Many of these recurrently mutated genes have been identified as likely false positives as they are generally more frequently mutated[3]. (B) Consensus driver prediction across four algorithms (Methods) indicating a lack of convincing evidence for novel SNV driver variants in coding or noncoding regions. SNV driver data are in Table S11 (Supplementary Dataset 1). All somatic variant calls predicted to disrupt protein coding genes are provided in Source Data.

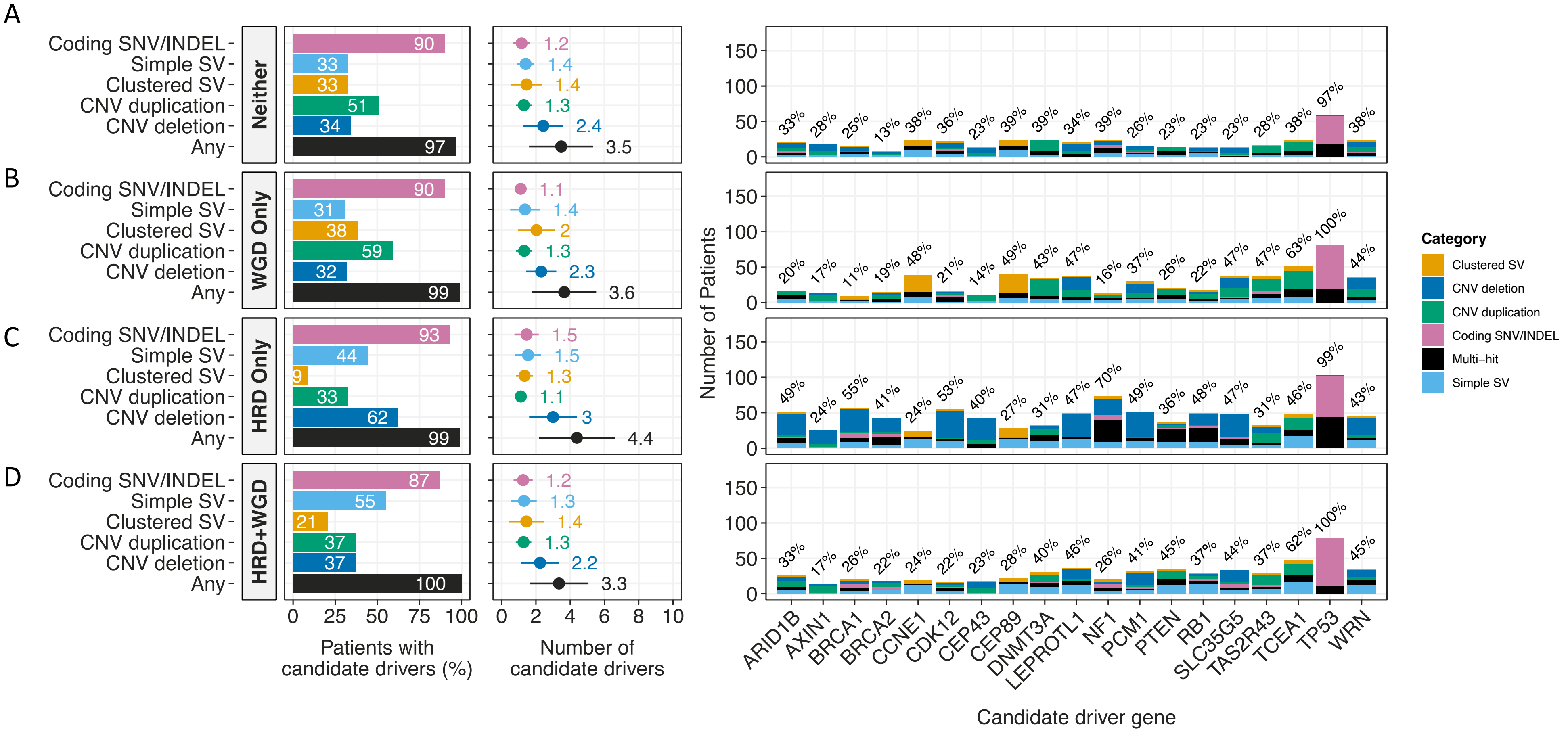

Supp Figure 8: Candidate driver landscape is robust to background genomic states of HRD and WGD. All figures are as described for Figure 3B and C but in a subset of samples. (A) Candidate driver landscape in tumours occupying 4 different classes: neither HRD nor WGD (N=61), (B) WGD only (N=81), (C) HRD only (N=104) and (D) both HRD and WGD (N=78). The forest plots represent the mean number of each type of driver mutation across tumours with at least one event and the standard deviation (whiskers), given the sample size in each class. All somatic variant calls predicted to disrupt protein coding genes are provided in Source Data.

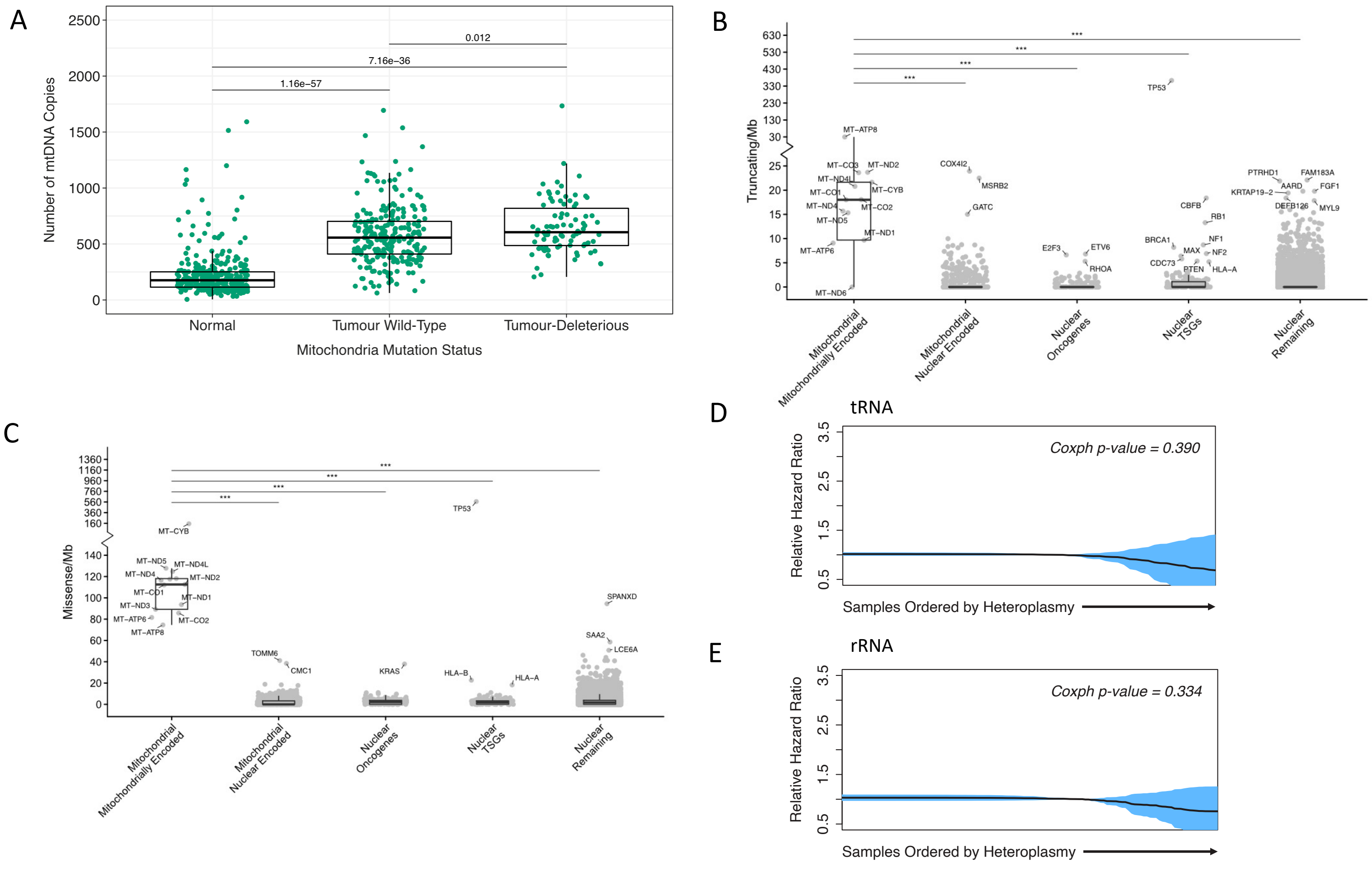

Supp Figure 9: High mitochondrial abundance and somatic mutation loads in HGSOC and the impact of tRNA and rRNA mutations on overall survival mediated by heteroplasmy. (A) Tumour samples have significantly higher mtDNA copy number than normal samples, and 26% (N=73) of samples in the cohort with survival data (N=277) carry predicted deleterious mtDNA SNVs (Table S13, Supplementary Dataset 1). Tumour samples show significant (Wilcoxon $p<0.001$) excesses of (B) truncating and (C) missense SNVs in mitochondrial genes versus in other gene classes of interest. Cox proportional hazards ratios for tRNA SNVs (D) and rRNA SNVs (E) are not significantly associated with overall survival (Methods) in samples with survival data (N=277) at any level of heteroplasmy. All box plots show the data median (centre), 25% and 75% percentiles (box boundaries), dispersal (whiskers indicating 1.5 times the interquartile range) and outliers (points). mtDNA SNV data are in Table S13 (Supplementary Dataset 1).

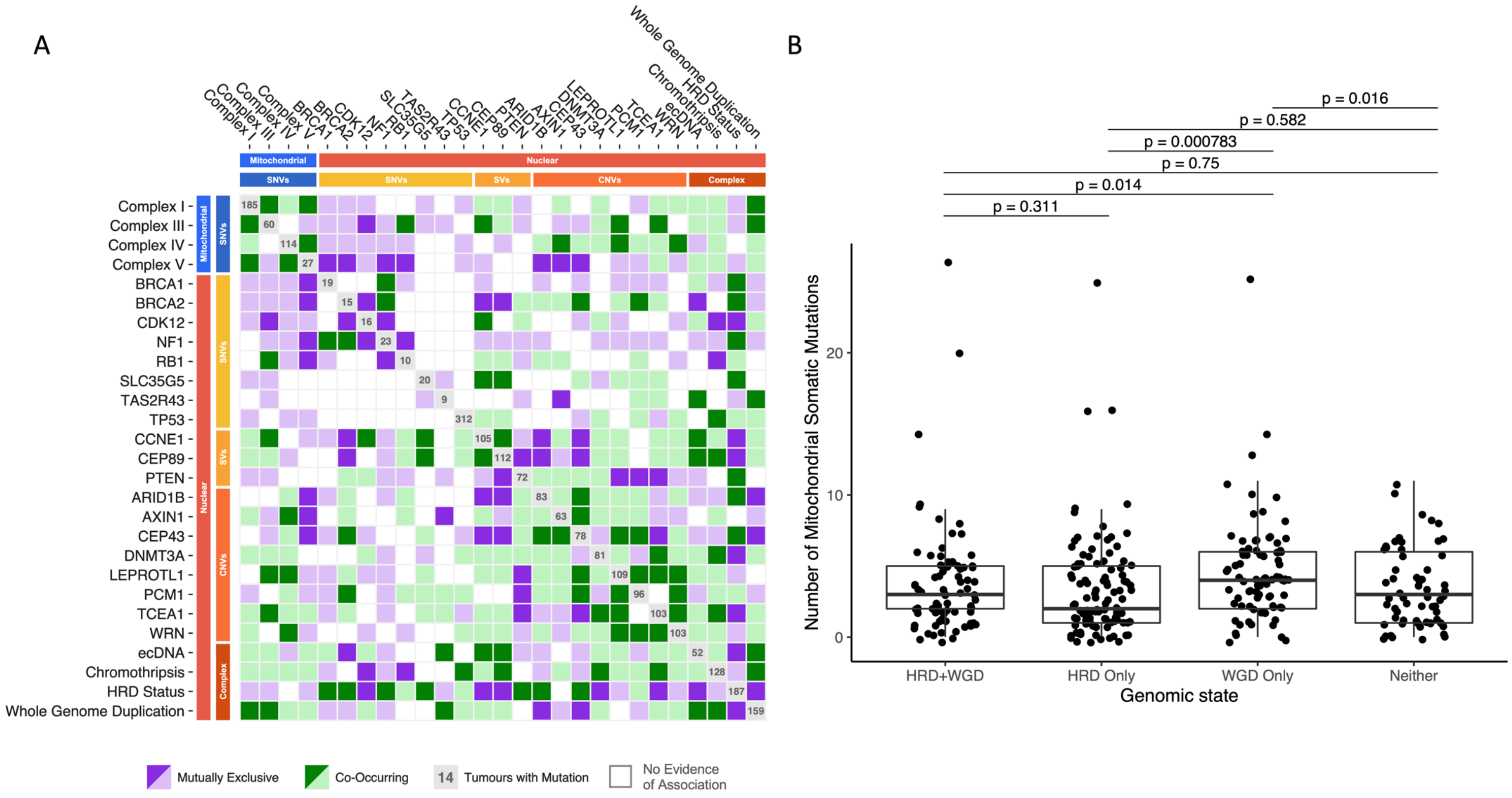

Supp Figure 10: Patterns of co-occurrence and mutual exclusivity between nuclear genome alterations and somatic mtDNA variants. (A) Evolutionary dependencies among nuclear and mtDNA somatic mutations using Bayesian inference[4] across N=324 HGSOC tumours, where significant co-occurrence (green) indicates synergistic interactions and mutually exclusivity (purple) indicates functional redundancies (p<0.05). (B) Relative enrichment of mtDNA mutations (Table S13 (Supplementary Dataset 1) in samples by background genomic state: both HRD and WGD (N=78), HRD only (N=104), WGD only (N=81), Neither (N=61). All box plots show the data median (centre), 25% and 75% percentiles (box boundaries), dispersal (whiskers indicating 1.5 times the interquartile range) and outliers (points). All somatic variant calls predicted to disrupt protein coding genes are provided in Source Data.

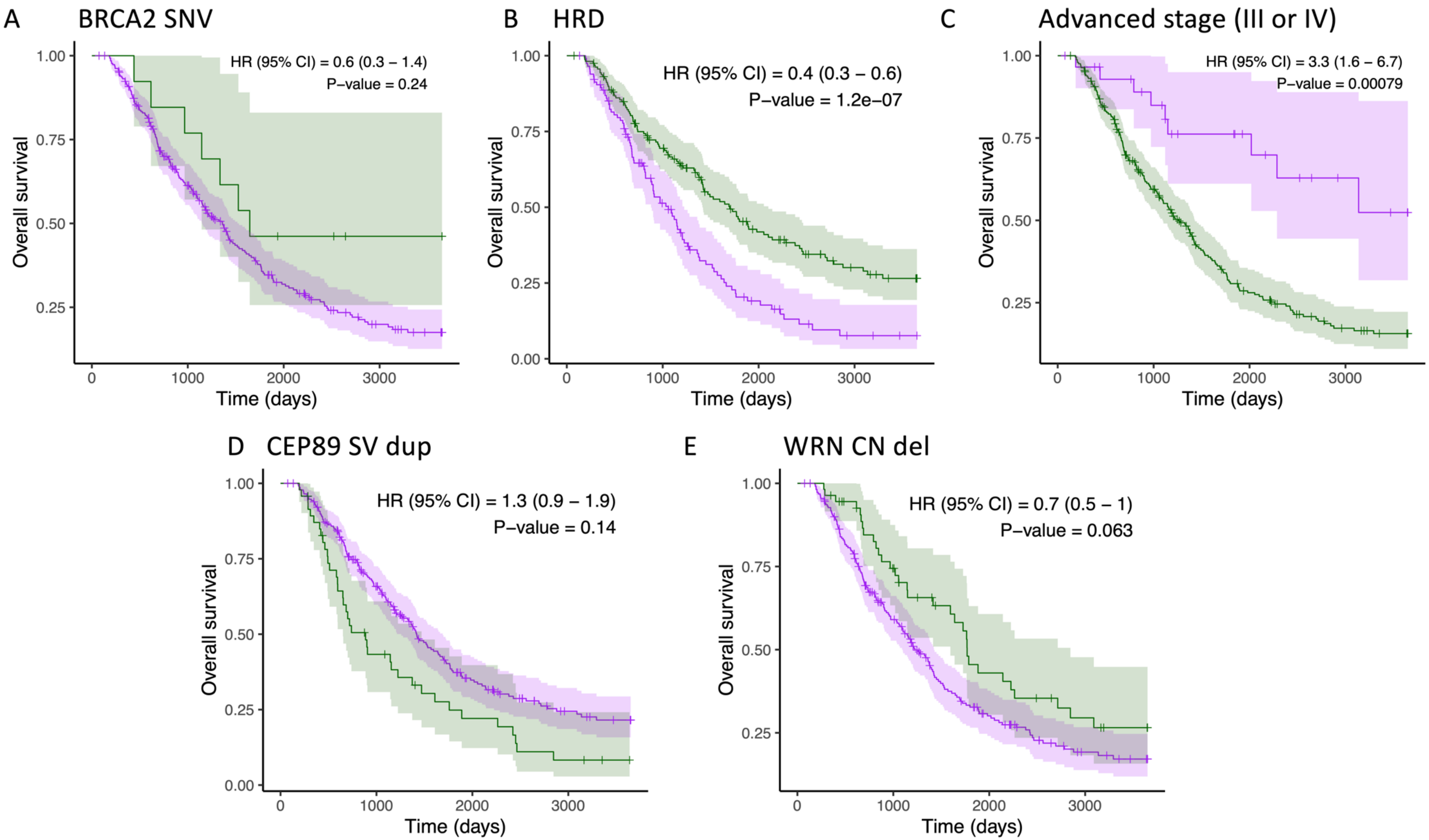

Supp Figure 11: Kaplan-Meier curves of presence (blue curve) and absence (red curve) of selected features (not shown in Figure 6) in the Cox proportional hazards elastic net model of overall survival (N=277) adjusting for the established effects of age, stage, and HRD status, stratifying by cohort. (A) BRCA2 SNV, (B) HRD, (C) stage at diagnosis (D) CEP89 SV duplication and (E) WRN CN deletion. CEP89 SV duplication is highly correlated with CCNE1 SV duplication and likely reflects the same signal, as attribution of effects in the presence of high correlation is arbitrary in the elastic net. Survival data are in Tables S12 and S14 (Supplementary Dataset 1).

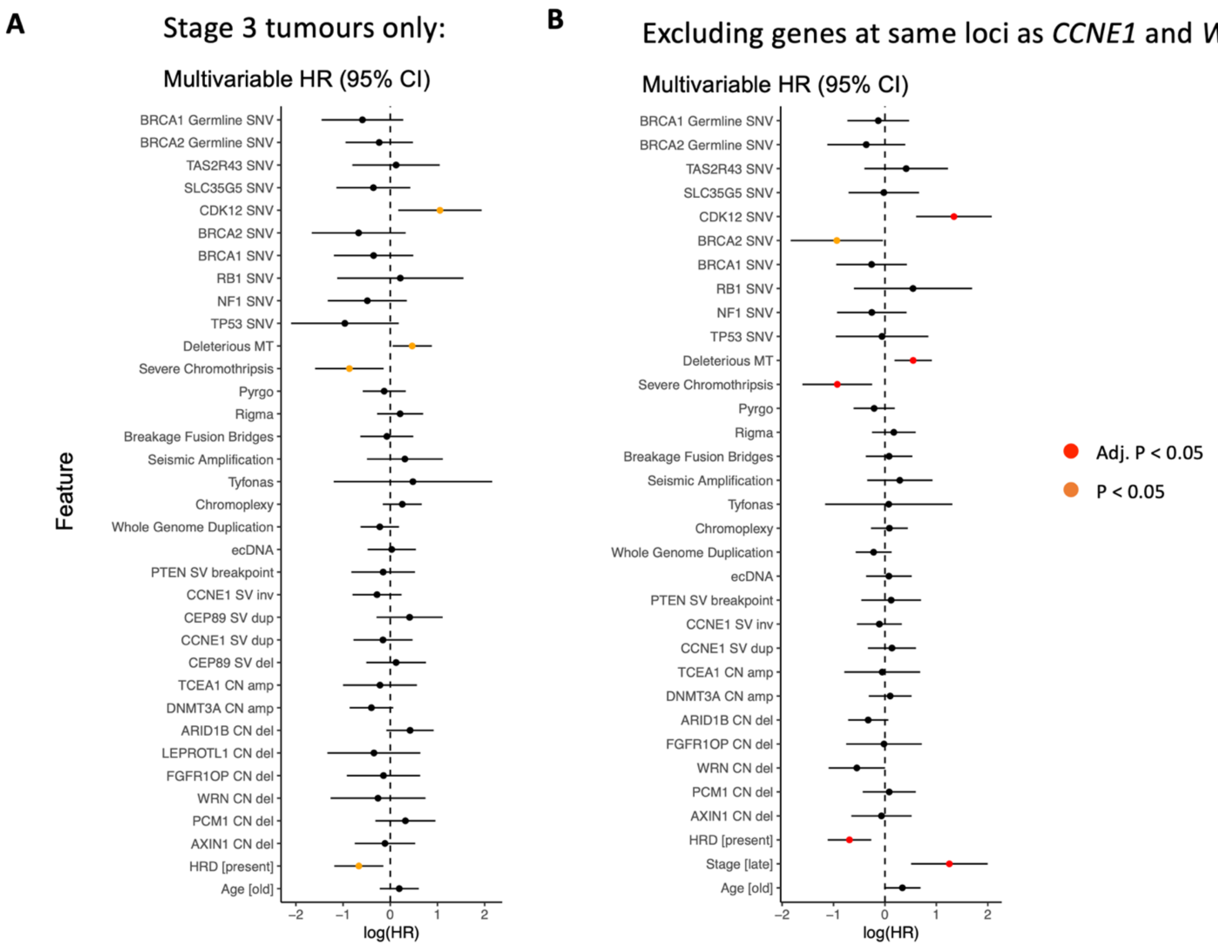

Supp Figure 12: A) Multivariable modelling of the impact of genomic features in HGSOC on overall survival adjusted for baseline clinical factors exclusively in tumours at stage 3 at diagnosis (N=248 of 324 tumours overall). Hazards ratios are derived from a multivariable model of 32 genomic features using a Cox Proportional hazards model adjusted for HRD and age at diagnosis, stratified by cohort (Methods). B) Multivariable modelling of the impact of the same genomic features, excluding events at CEP89 and LEPROTL1 which are near to CCNE1 and WRN respectively, on overall survival (N=277) adjusted for baseline clinical factors. Hazards ratios are derived from a multivariable model of 30 genomic features using a Cox Proportional hazards model adjusted for HRD, stage and age at diagnosis, stratified by cohort. Forest plots show hazards ratios (log) and 95% confidence interval per feature. Survival data are in Tables S12 and S14 (Supplementary Dataset 1).

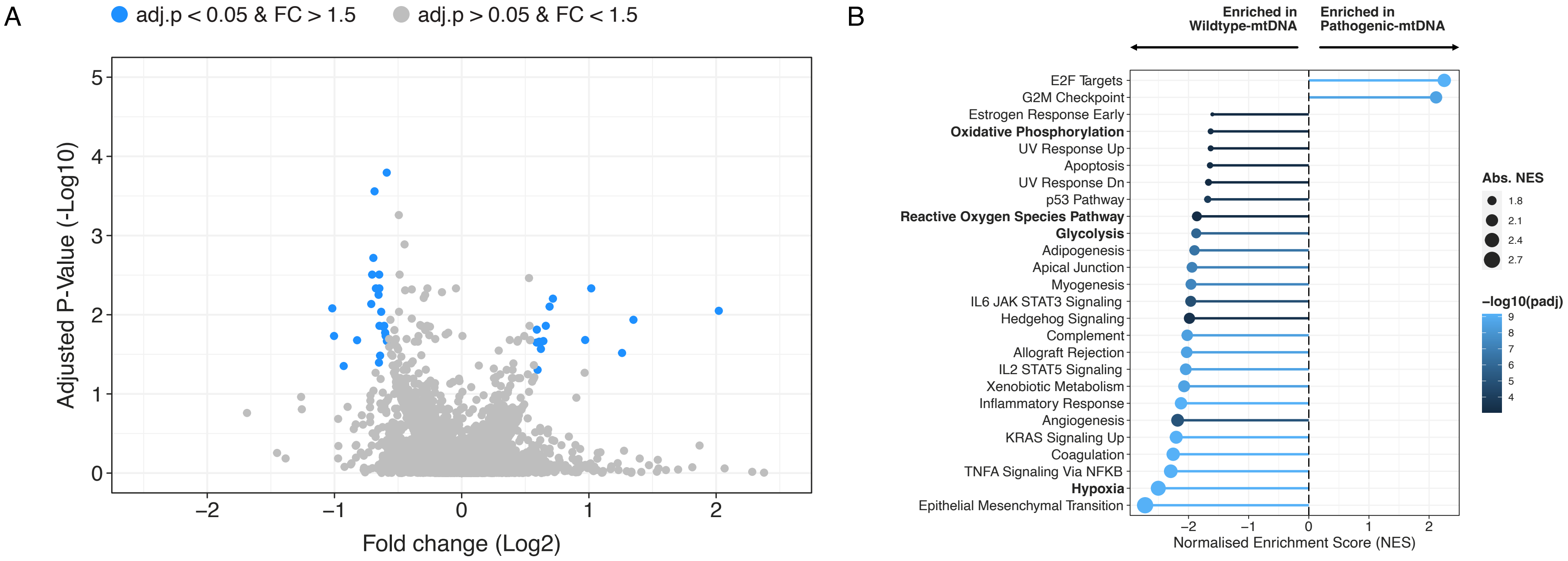

Supp Figure 13: Pathogenic mtDNA variants are associated with differential expression. (A) Volcano plot depicting the differential expression between HGSOC tumour samples with and without pathogenic mtDNA variants, indicating (blue points) only 47 significantly differentially expressed (DE) genes across the mitochondrial and nuclear genomes. (B) Gene set enrichment analysis using the GSEA tool[5] comparing the ranked genes of tumours with and without pathogenic mtDNA variants, reveals pathways enriched ($p < 0.001$) in each group of tumours. Genes were ranked based on the DESeq2 test statistic and GSEA was performed using the fGSEA R package (v1.16.0) with a minimum gene set size of 10, a maximum of 500 genes, and 100,000 permutations, against the MSigDB Hallmark gene set collection[6]. mtDNA SNV data are in Table S13 (Supplementary Dataset 1).

Supplementary References

1. Drews RM, Hernando B, Tarabichi M, Haase K, Lesluyes T, Smith PS, Morrill Gavarró L, Couturier DL, Liu L, Schneider M, Brenton JD, Van Loo P, Macintyre G, Markowetz F. A pan-cancer compendium of chromosomal instability. Nature. 2022. 606:976-983. doi: https://doi.org/10.1038/s41586-022-04789-9
2. Espejo Valle-Inclán J, Cortés-Ciriano I. ReConPlot: an R package for the visualization and interpretation of genomic rearrangements. Bioinformatics. 2023. 39:btad719. doi: https://doi.org/10.1093/bioinformatics/btad719
3. Shyr C, Tarailo-Graovac M, Gottlieb M, Lee JJ, van Karnebeek C, Wasserman WW. FLAGS, frequently mutated genes in public exomes. BMC Med Genomics. 2014. 7:64. doi: https://doi.org/10.1186/s12920-014-0064-y
4. Mina M, Iyer A, Tavernari D, Raynaud F, Ciriello G. Discovering functional evolutionary dependencies in human cancers. Nat Genet. 2020. 52:1198-1207. doi: https://doi.org/10.1038/s41588-020-0703-5
5. Subramanian A, Tamayo P, Mootha VK, Mukherjee S, Ebert BL, Gillette MA, Paulovich A, Pomeroy SL, Golub TR, Lander ES, Mesirov JP. Gene set enrichment analysis: a knowledge-based approach for interpreting genome-wide expression profiles. Proc Natl Acad Sci U S A. 2005. 102:15545-50. doi: https://doi.org/10.1073/pnas.0506580102
6. Liberzon A, Birger C, Thorvaldsdóttir H, Ghandi M, Mesirov JP, Tamayo P. The Molecular Signatures Database (MSigDB) hallmark gene set collection. Cell Syst. 2015. 1:417-425. doi: https://doi.org/10.1016/j.cels.2015.12.004

Scottish Genomes Partnership Consortium

Timothy J. Aitman[1], Andrew V. Biankin[2,3,4], Susanna L. Cooke[2], Wendy Inglis Humphrey[1], Sancha Martin[2], Lynne Mennie[5], Alison Meynert[6], Zosia Miedzybrodzka[5], Fiona Murphy[8], Craig Nourse[2], Javier Santoyo-Lopez[9], Colin A. Semple[6], and Nicola Williams[10]

1. Centre for Genomic and Experimental Medicine, MRC Institute of Genetics and Cancer, University of Edinburgh, Edinburgh, UK
2. School of Cancer Sciences, Wolfson Wohl Cancer Research Centre, University of Glasgow, Glasgow, UK
3. West of Scotland Pancreatic Unit, Glasgow Royal Infirmary, Glasgow, UK
4. South Western Sydney Clinical School, Faculty of Medicine, University of NSW, Liverpool NSW 2170, Australia
5. Clinical Genetics Centre, Ashgrove House Aberdeen Royal Infirmary, Foresterhill, Aberdeen, UK
6. MRC Human Genetics Unit, Institute of Genetics and Cancer, University of Edinburgh, Edinburgh, UK
7. Clinical Genetics Centre, Ashgrove House Aberdeen Royal Infirmary, Foresterhill, Aberdeen, UK
8. National Services Division, NHS National Services Scotland, Gyle Square, 1 S Gyle Cres, Edinburgh, UK

9. Edinburgh Genomics, Ashworth Laboratories, The King's Buildings, Charlotte Auerbach Rd, Edinburgh, UK
10. West of Scotland Centre for Genomic Medicine, Laboratory Genetics, Queen Elizabeth University Hospital, Glasgow, UK
