# Supplementary material for: Divergent trajectories to structural diversity impact patient survival in high grade serous ovarian cancer
Source: Nat Commun. 2025 Jul 1;16:5586. doi: 10.1038/s41467-025-60655-y (PMC12215056; doi:10.1038/s41467-025-60655-y)
Supplement: Supplementary file 3 — Reporting Summary [file 41467_2025_60655_MOESM3_ESM.pdf]

nature portfolio

Corresponding author(s): Ailith Ewing

Last updated by author(s): May 15, 2025

Reporting Summary

Nature Portfolio wishes to improve the reproducibility of the work that we publish. This form provides structure for consistency and transparency in reporting. For further information on Nature Portfolio policies, see our Editorial Policies and the Editorial Policy Checklist.

Statistics

For all statistical analyses, confirm that the following items are present in the figure legend, table legend, main text, or Methods section.

n/a	Confirmed	
☐	☒	The exact sample size (*n*) for each experimental group/condition, given as a discrete number and unit of measurement
☐	☒	A statement on whether measurements were taken from distinct samples or whether the same sample was measured repeatedly
☐	☒	The statistical test(s) used AND whether they are one- or two-sided<br>*Only common tests should be described solely by name; describe more complex techniques in the Methods section.*
☐	☒	A description of all covariates tested
☐	☒	A description of any assumptions or corrections, such as tests of normality and adjustment for multiple comparisons
☐	☒	A full description of the statistical parameters including central tendency (e.g. means) or other basic estimates (e.g. regression coefficient) AND variation (e.g. standard deviation) or associated estimates of uncertainty (e.g. confidence intervals)
☐	☒	For null hypothesis testing, the test statistic (e.g. *F*, *t*, *r*) with confidence intervals, effect sizes, degrees of freedom and *P* value noted<br>*Give P values as exact values whenever suitable.*
☒	☐	For Bayesian analysis, information on the choice of priors and Markov chain Monte Carlo settings
☒	☐	For hierarchical and complex designs, identification of the appropriate level for tests and full reporting of outcomes
☐	☒	Estimates of effect sizes (e.g. Cohen's *d*, Pearson's *r*), indicating how they were calculated

Our web collection on statistics for biologists contains articles on many of the points above.

Software and code

Policy information about availability of computer code

Data collection	All code for manuscript available here: https://github.com/EwingGroup/TrajectoriesHGSOC. Primary processing code used in previous publication and this one is here: https://github.com/ailithewing/Structural_variants_BRCA1_2_HRD_inHGSOC
Data analysis	All code for manuscript available here: https://github.com/EwingGroup/TrajectoriesHGSOC.

For manuscripts utilizing custom algorithms or software that are central to the research but not yet described in published literature, software must be made available to editors and reviewers. We strongly encourage code deposition in a community repository (e.g. GitHub). See the Nature Portfolio guidelines for submitting code & software for further information.

Data

Policy information about availability of data

All manuscripts must include a data availability statement. This statement should provide the following information, where applicable:

- Accession codes, unique identifiers, or web links for publicly available datasets
- A description of any restrictions on data availability
- For clinical datasets or third party data, please ensure that the statement adheres to our policy

All WGS and RNA-seq data are available from the European Genome/Phenome Archive (EGA) and the US National Cancer Institute (NCI) as follows. Australian Ovarian Cancer Study (AOCS)8: EGAD00001000293 [https://www.ega-archive.org/studies/EGAS00001000397], British Colombia Cancer Agency (BCCA)23: EGAD00001003268 [https://www.ega-archive.org/datasets/EGAD00001003268], MD Anderson (MDA)25: EGAD00001005240 [https://ega-archive.org/dacs/EGAC00001001288] are available under restricted access due to data privacy laws; access can be requested from their respective Data Access Committees via EGA.

The Cancer Genome Atlas (TCGA)6: NCI Genomic Data Commons [https://portal.gdc.cancer.gov/]. The clinical information for the AOCS and TCGA patients is available as part of the PCAWG project 41. Clinical information for the MDA and BCCA cohorts are available from the supplementary data of their respective publications 23,25. All SHGSOC cohort data (WGS and RNA-seq) generated for this study are available at accession number EGAS00001004410 [https://www.ega-archive.org/studies/EGAS00001004410] under restricted access due to (UK GDPR) data privacy laws. Access can be arranged for researchers for 12 months by a simple request to the Data Access Committee (DAC) via EGA; the DAC will reply to requests within one week.

Research involving human participants, their data, or biological material

Policy information about studies with human participants or human data. See also policy information about sex, gender (identity/presentation), and sexual orientation and race, ethnicity and racism.

Reporting on sex and gender	Findings only applicable to people with ovaries. All participants female sex.
Reporting on race, ethnicity, or other socially relevant groupings	Not included in this study.
Population characteristics	All participants have high grade serous ovarian cancer.
Recruitment	Patients recruited in ovarian cancer clinics participating in their respective studies. The previously published AOCS study is enriched for patients with chemo resistant/ refractory disease.
Ethics oversight	Ethics of previously published studies e.g. AOCS, BCCA, MDA and TCGA covered by them. SHGSOC study covered by Lothian Annotated Human BioResource (ethics reference 15/ES/0094-SR751) and NHS Greater Glasgow & Clyde Biorepository (ethics reference 22/WS/0020).

Note that full information on the approval of the study protocol must also be provided in the manuscript.

Field-specific reporting

Please select the one below that is the best fit for your research. If you are not sure, read the appropriate sections before making your selection.

[x] Life sciences   [ ] Behavioural & social sciences   [ ] Ecological, evolutionary & environmental sciences

For a reference copy of the document with all sections, see nature.com/documents/nr-reporting-summary-flat.pdf

Life sciences study design

All studies must disclose on these points even when the disclosure is negative.

Sample size	324 high grade serous ovarian cancer patients. This is the largest number that we could assemble and recruit within the timescale of the study.
Data exclusions	Samples excluded if proportion of tumour cells in the sample was too low or pathological/clinical/genomic review suggested that the tumour was not high grade serous ovarian.
Replication	Findings replicated in 5 independently collected ovarian cancer cohorts.
Randomization	N/A. Patients assigned to groups according to their tumour characteristics.
Blinding	Blinding not relevant for this study.

Reporting for specific materials, systems and methods

We require information from authors about some types of materials, experimental systems and methods used in many studies. Here, indicate whether each material, system or method listed is relevant to your study. If you are not sure if a list item applies to your research, read the appropriate section before selecting a response.

Materials & experimental systems

n/a	Involved in the study	
[x]	[ ]	Antibodies
[x]	[ ]	Eukaryotic cell lines
[x]	[ ]	Palaeontology and archaeology
[x]	[ ]	Animals and other organisms
[ ]	[x]	Clinical data
[x]	[ ]	Dual use research of concern
[x]	[ ]	Plants

Methods

n/a	Involved in the study	
[x]	[ ]	ChIP-seq
[x]	[ ]	Flow cytometry
[x]	[ ]	MRI-based neuroimaging

Clinical data

Policy information about clinical studies

All manuscripts should comply with the ICMJE guidelines for publication of clinical research and a completed CONSORT checklist must be included with all submissions.

Clinical trial registration	Not a clinical trial.
Study protocol	Not a clinical trial.
Data collection	Clinical data available via Lothian Annotated Human BioResource (ethics reference 15/ES/0094-SR751) and NHS Greater Glasgow & Clyde Biorepository (reference 22/WS/0020).
Outcomes	Research purpose was discovery rather than confirmatory. All results adjusted for multiple testing.

Plants

Seed stocks	N/A
Novel plant genotypes	N/A
Authentication	N/A
